# The complete chloroplast genome of Lilium rosthornii Diels (Liliopsida: Liliaceae) from Hunan, China

**DOI:** 10.1080/23802359.2021.1872452

**Published:** 2021-02-14

**Authors:** Hongzhi Wu, Weiwei Bai, Zijie Li, Shuilian He, Wenbin Yuan, Jingzhi Wu

**Affiliations:** Yunnan Agricultural University, Kunming, China

**Keywords:** *Lilium rosthornii*, chloroplast genome, genome sequence

## Abstract

*Lilium rosthornii* is the perennial herbaceous bulbous plant belonging to the Lily of the Liliaceae, with high ornamental value and medicinal values. In this present study, we sequenced the complete chloroplast genome of *Lilium rosthornii* by Illumina Hiseq X Ten and PacBio RS technologies firstly. The genome size of *L. rosthornii,* was 152,242bp, with typical tetragonal structure: one large single-copy (LSC, 81,875 bp), one small single-copy (SSC, 17,553 bp), and a pair of inverted repeat regions (IRs, 26,407 bp). The overall GC content was 37.02%. The complete genome contained 131 genes, including 85 protein-coding genes, 38 tRNA genes, and eight rRNA genes. Phylogenetic analysis placed *L. rosthornii* under the family Liliaceae.

*Lilium rosthornii* belongs to perennial herb of Liliaceae, which is a wild specie and rarely cultivated. It originated from the gullies, streams and forests around Jinfo Mountain, Nanchuan City, Chongqing, with an altitude of 350–1100 m. The flowering period of *L. rosthornii* is from June to September. Dried fleshy scales are often used as medicinal materials, which has the excellent efficacy in the treatment of lung diseases, health preserving and enriching blood. The *L. rosthornii* as an perennial herbaceous bulbous plant with high ornamental value (Liu et al 2006). It has a long flowering period, the petals are usually reddish yellow or yellow. Besides its flower type feels like *Lilium lancifolium*, the perianth has purple spots and its texture is smooth.

Vegetative production is an effective method to preserve germplasm and utilize plant traits of *L. rosthornii,* which is an important resource of Liliaceae biodiversity, at the same time it is a significant breeding material in horticulture (Wang and Sha 2008). Due to the large-scale collection, its wild resources have been seriously damaged. The success of tissue culture and rapid propagation system provides technology and way for the protection and sustainable utilization of this wild species. In the present study, we determined the chloroplast genome sequence of *Lilium rosthornii*, and discussed the genetic relationship among various species in Liliaceae.

The complete genomic DNA was extracted from fresh plant leaves and *Lilium rosthornii* was collected from the national natural reserve of Hupin mountain in Hunan province. Additional specimens were kept in Herbarium of Yunnan Agricultural University under the collection number 2020WHZ002. Total genomic DNA was isolated from fresh leaves using a DNeasy Plant Mini Kit (QIAGEN, Valencia, California, USA) according to the manufacturer’s instructions to construction chloroplast DNA libraries. Sequencing was carried out on an Illumina NovaSeq platform. The output was a 5 Gb raw data of 150 bp paired end reads, further trimmed and assembled using SPAdes (Bankevich et al. 2012). Resultant clean reads were assembled using GetOrganelle pipeline (https://github.com/Kinggerm/GetOrganelle). Annotations of chloroplast genome were conducted by the software Geneious (Kearse et al.^2012^) and checked by comparison against the *Lilium taliense* complete chloroplast genome (GenBank accession number: KY009938).

The results of whole genome sequencing showed that the size of chloroplast genome of *L. rosthornii* (Genbank accession number: MW136390) was 152,242 bp with a typical tetragonal structure: one large single-copy (LSC, 81,875 bp), one small single-copy (SSC, 17,553 bp) and two reverse repeats (IRS, 26,407 bp). The total GC content was 37.0%, the GC content of large single copy (LSC) was 34.8%, and the GC content of small single copy (SSC) was 30.6%. A total of 131 genes were detected, including 85 protein-coding genes, 38 tRNA genes and 8 rRNA genes. Nineteen gene are partially or completely duplicated, including seven PCG (rpl2; rpsl23; ycf2; ndhB; rps12; rps7; ycf1), eight tRNA (trnI-GAU, trnA-UGC, trnL-CAA, trnI-CAU, trnR-ACG, trnV-GAC, trnN-GUU, trnH-GUG) and all four rRNA (4.5S, 5S, 16S & 23S rRNA). All the rRNA genes in the genome sequence were located in the repeat region.

Phylogenetic analysis was constructed based on the complete chloroplast genome sequence obtained from *L. rosthornii* with those of 25 reported species in the Lilium genus and four out-groups using the maximum likelihood (ML) analysis by RAxML 8.0 software (Stamatakis 2014). The maximum likelihood tree was constructed with 1000 bootstrap replicates using FastTree software (Liu et al. 2011). According to the result of the analysis, *L. rosthornii* belongs to family Liliaceae and is associated with other *Lilium* species. The taxonomic status of *L. rosthornii* exhibits a closest relationship with *L. taliense* and *L. bakerianum*. This finding could serve as valuable genomic resources providing insight into conservation and exploitation efforts for this medicinal species. (Figure 1).

**Figure 1.**
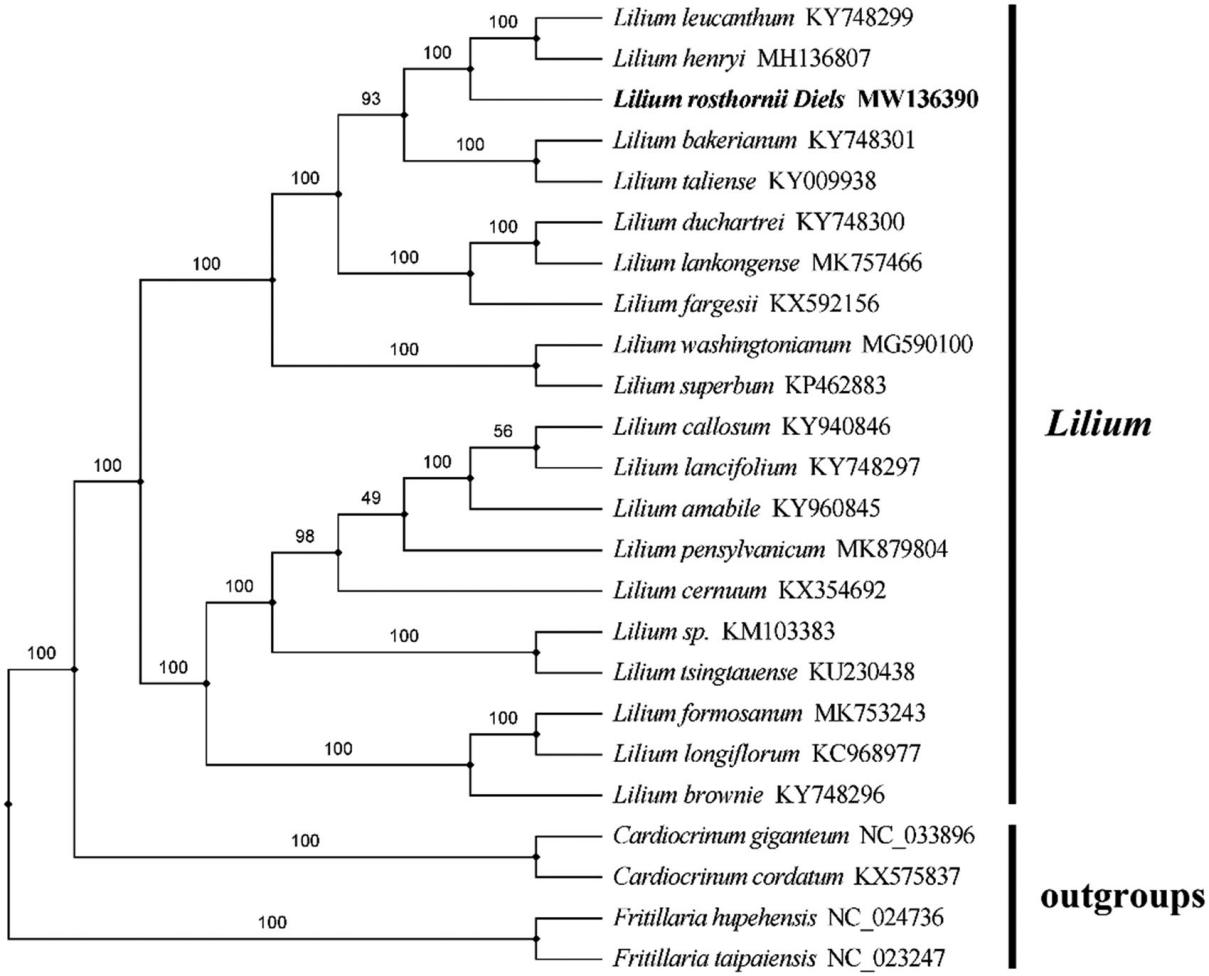
A phylogenetic tree of the *Lilium* species based on the completed chloroplast genomes of 25 species and 4 outgroup species. we downloaded all the other sequences from NCBI GenBank.

## Data Availability

The data that support the findings of this study are openly available in GenBank of NCBI at https://www.ncbi.nlm.nih.gov/, reference number MW136390.
